# Assessing the documentation of publicly available medical image and signal datasets and their impact on bias using the BEAMRAD tool

**DOI:** 10.1038/s41598-024-83218-5

**Published:** 2024-12-30

**Authors:** Maria Galanty, Dieuwertje Luitse, Sijm H. Noteboom, Philip Croon, Alexander P. Vlaar, Thomas Poell, Clara I. Sanchez, Tobias Blanke, Ivana Išgum

**Affiliations:** 1 Informatics Institute, University of Amsterdam, Amsterdam, The Netherlands; 2 Department of Biomedical Engineering and Physics, Amsterdam UMC location University of Amsterdam, Amsterdam, The Netherlands; 3 Department of Media Studies, Faculty of Humanities, University of Amsterdam, Amsterdam, The Netherlands; 4 Department of Intensive Care, Amsterdam UMC, University of Amsterdam, Amsterdam, The Netherlands; 5 Department of Cardiology, Amsterdam UMC location University of Amsterdam, Amsterdam, The Netherlands; 6 Department of Radiology and Nuclear Medicine, Amsterdam UMC location University of Amsterdam, Amsterdam, The Netherlands; 7 Section of Cardiovascular Medicine, Department of Internal Medicine, Yale School of Medicine, New Haven, CT, United States

**Keywords:** Health care, Computer science, Scientific data

## Abstract

Medical datasets are vital for advancing Artificial Intelligence (AI) in healthcare. Yet biases in these datasets on which deep-learning models are trained can compromise reliability. This study investigates biases stemming from dataset-creation practices. Drawing on existing guidelines, we first developed a BEAMRAD tool to assess the documentation of public Magnetic Resonance Imaging (MRI); Color Fundus Photography (CFP), and Electrocardiogram (ECG) datasets. In doing so, we provide an overview of the biases that may emerge due to inadequate dataset documentation. Second, we examine the current state of documentation for public medical images and signal data. Our research reveals that there is substantial variance in the documentation of image and signal datasets, even though guidelines have been developed in medical imaging. This indicates that dataset documentation is subject to individual discretionary decisions. Furthermore, we find that aspects such as hardware and data acquisition details are commonly documented, while information regarding data annotation practices, annotation error quantification, or data limitations are not consistently reported. This risks having considerable implications for the abilities of data users to detect potential sources of bias through these respective aspects and develop reliable and robust models that can be adapted for clinical practice.

**Supplementary Information:**

The online version contains supplementary material available at 10.1038/s41598-024-83218-5.

## Introduction

Medical datasets constitute valuable resources for researchers as they enable data-driven scientific innovation and provide deeper insights into patients’ disease and their trajectories. Ideally, these datasets should offer a foundation for training and testing trustworthy deep-learning applications. However, previous studies have demonstrated that these models trained on medical data may encode various biases—i.e., systematic errors in deep learning models affecting the models’ ability to “classify subgroups of patients, estimate risk levels, or make predictions”^1^. These biases may already be present in the datasets specific models are trained on, or models may introduce new types due to unknown confounders^2–4^.

The notion of bias has been highlighted in various medical domains, including radiology, ophthalmology, and cardiology^5–7^. As Abbasi-Sureshjani et al.^8^ explain, bias may emerge because of insufficient consideration of demographic variables such as age, sex, and race in the construction of medical datasets used to train machine learning models. In addition, scholars have argued that bias may be introduced due to inconsistencies in inclusion criteria or patterns in missing data in a dataset^9,10^. This can have significant implications for model outcomes as they risk dropping in performance on societal subgroups whose data are underrepresented in a training set^4,8,11,12^.

To detect and mitigate types of dataset biases, scholars have called for thorough and standardized dataset documentation^9,11,13,14^, which led to the introduction of datasheets^13,15^. Datasheets are documents that contain essential dataset information, including motivation, composition, collection process, and recommended use. While the content of datasheets will vary depending on the domain and specific characteristics of a dataset, their creators and users can benefit from comprehensive documentation in four ways. First, datasheets have the “potential to increase transparency and accountability” within AI research communities^15^. Second, they allow dataset creators and users to detect and mitigate unwanted societal biases in deep learning models and facilitate greater reproducibility of their deep learning results. Third, datasheets help “researchers and practitioners to select more appropriate datasets for their chosen tasks”^15^. Lastly, datasheets allow dataset creators to critically reflect on data collection, distribution, and maintenance processes. Following Rostamzadeh et al., such reflections are particularly important in the medical context as they promote transparency, thoroughness, and lead to more reliable models^13^. A lack of transparency in dataset reporting, in turn, makes it more difficult to assess the risk of bias on models trained on that data^11^. This for example becomes clear in the study by Roberts et al., who show that the lack of awareness on the use of datasets constructed from various overlapping data sources leads to the emergence of various biases after training^16^.

In medical imaging research, the development of dataset documentation guidelines has centered around the organization of image analysis challenges. The primary objective of the challenges involves the assessment of different research approaches to address a specific image analysis problem on identical datasets to identify the best-performing methods. However, recent scholarship has highlighted that common design practices in these challenges, including dataset design and documentation, vary significantly and lack standardized reporting^17^. This makes it increasingly difficult to adequately interpret and facilitate the reproducibility of challenge results^17^. In response, the **B**iomedical **I**mage **A**nalysis Challenge**S** (BIAS) initiative founded by the challenge working group of the Medical Image Computing and Computer Assisted Intervention (MICCAI) Society, developed a set of recommendations for the reporting of challenges in 2018^18^. By now, these guidelines are recognized as a valuable resource for enhancing the quality of challenge design, including aspects such as dataset construction and documentation^11,18^.

In light of these observations, this paper qualitatively investigates and reviews the documentation of publicly available imaging datasets and other data types. To do so, we draw from existing documentation guidelines and developed a Bias Evaluation And Monitoring for Transparent And Reliable Medical Datasets (BEAMRAD) tool for comprehensive dataset documentation evaluation to closely assess the documentation of (challenge-related) public medical imaging datasets that have been released after the publication of the BIAS protocol^18^. Here, we specifically review the documentation of two types of imaging data datasets—Magnetic Resonance Imaging (MRI) and Color Fundus Photography (CFP). These modalities have been selected as examples for the application of our BEAMRAD evaluation tool, with MRI being one of the central image modalities for which deep-learning models are being developed^19,20^. To also consider imaging data available in other clinical fields, CFP datasets have been selected as this data is one of the most used and widely adopted imaging modalities outside radiology, present in hospitals, private or public eye clinics, large screening sites, and opticians^21,22^. The size of these datasets and the fact that the data is often collected at multiple locations using a variety of different scanners is considered to challenge the diversity and the quality of the acquired data^23^. These qualitative assessments are important as little is known about the ways in which existing guidelines are being followed and interpreted for the development of contemporary medical imaging datasets at scale. Such information, however, is vital considering the growing importance of public data for the development of deep-learning models in medical imaging and researchers’ ability to detect the potential sources of bias in these datasets through high-quality documentation.

In comparison to existing documentation tools and reporting guidelines, the BEAMRAD tool also allows us to thoroughly assess the documentation of publicly available datasets with medical signals—i.e. Electrocardiograms (ECGs). Like in imaging^24^, signal data is increasingly analyzed with deep-learning models using publicly available datasets^25–27^. Yet, according to our knowledge, little attention has been paid to the documentation of these datasets for this deep-learning subfield that does not rely on guidelines for dataset development and reporting such as BIAS for imaging data. Therefore, we contribute to this research through a critical assessment of the documentation accompanying large public ECG datasets and examine how particular forms of bias can emerge from these datasets.

In this paper, we thus present the BEAMRAD dataset documentation evaluation tool and a qualitative review of state-of-the-art publicly available medical image (MRI and CFP) and signal (ECG) dataset reporting. This tool supports the documentation of medical datasets in a way that facilitates the prevention, identification, and mitigation of potential biases arising from dataset construction. Through these assessments, we contribute to research on dataset production and documentation in AI ethics in medical image and signal analysis and shed light on the ways in which dataset documentation impacts the detection and mitigation of various biases in deep-learning models for these respective areas. As such, we show how documentation practices further impact the responsible use and re-use of deep-learning solutions in AI-based medical imaging and signal domains.

## Methodology

In this section, we explain the methods we developed to conduct this study in three stages. First, we describe the sections of the BEAMRAD tool. This includes describing the motivation behind the construction of the document. Second, we provide the criteria and motivations for the selection of the datasets. Third, we describe the method for the evaluation of the selected dataset documentation.

### Developing a tool for the evaluation of ML dataset documentation

To evaluate the documentation of medical image and signal datasets for the development and evaluation of machine learning models, we first developed the BEAMRAD tool—based on a ‘questionnaire’—that can be used for the thorough evaluation of datasets of various medical data types, including image and signal data (Appendix I). This tool specifically draws from existing dataset documentation tools and guidelines, including Datasheets for Datasets^15^, BIAS^18^, and the Critical Appraisal and Data Extraction for Systematic Reviews of Prediction Modelling Studies (CHARMS) checklist^10^. Such descriptions document the “motivation, composition, data collection processes and recommended use cases”, facilitating communication between dataset creators and dataset consumers to surface bias in datasets and encourage the prioritization of dataset transparency and accountability^26,28^. The BIAS challenge reporting guidelines have been selected as they are recognized as a key resource for enhancing the quality of dataset development and reporting for challenges in biomedical imaging^11,18^. The CHARMS guideline was not specifically designed for the evaluation of datasets or their documentation, but for reviewing machine-learning model development processes in their entirety^10^. It was selected as a large part of the document includes questions that focus on reviewing information from data used to train those models. Together, the BIAS and CHARMS guidelines have proven to be valuable for the evaluation of datasets in biomedical imaging^11^.

**Fig. 1:**
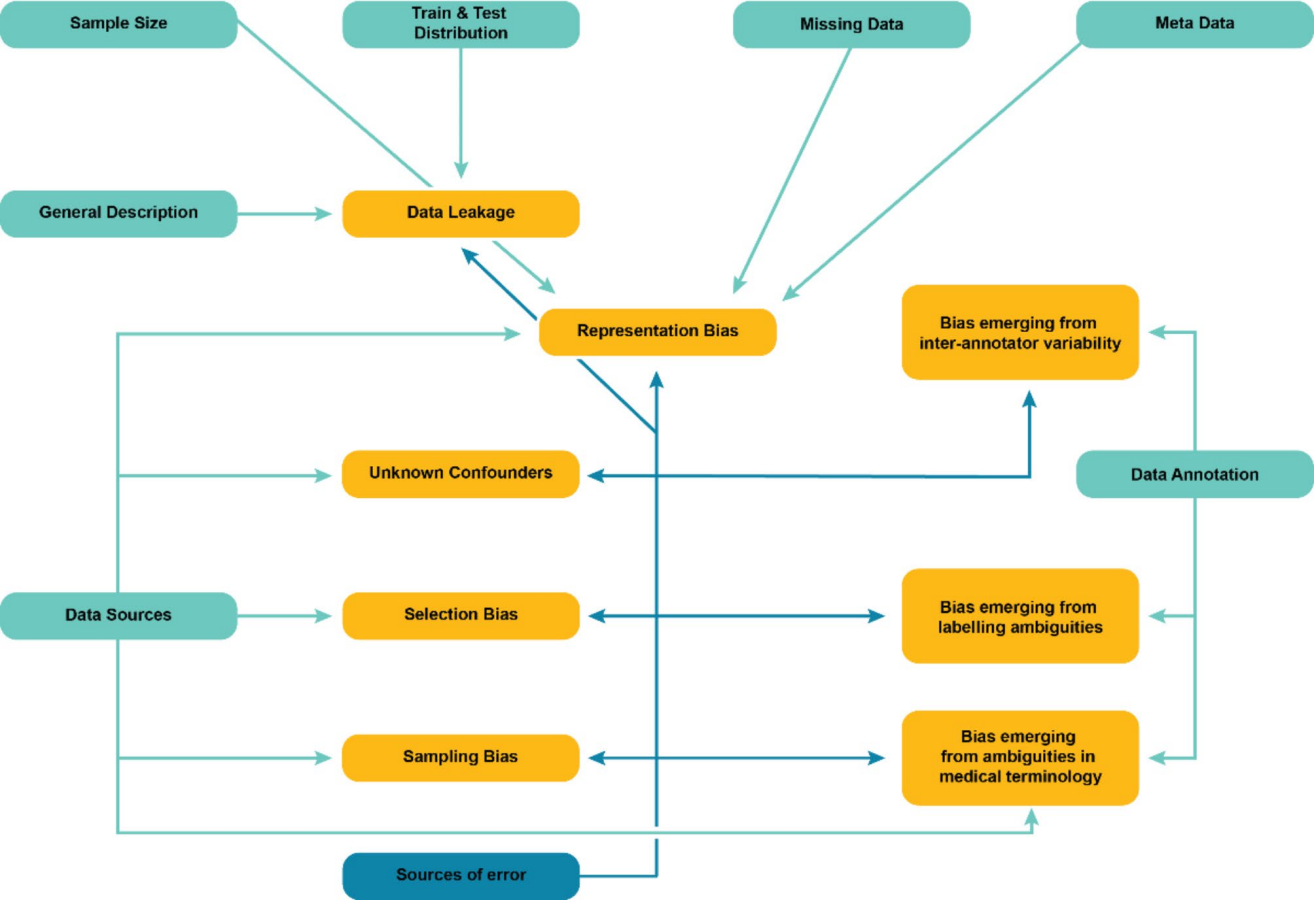
Overview of the potential bias implications for individual sections of the BEAMRAD tool developed for this study. The arrows connect dataset evaluation tool sections [blue rectangles] with potential biases [yellow rectangles] that may arise and become difficult to detect without clear documentation of specific dataset elements. The use of a distinct color for the error sources’ arrow emphasizes the importance of comprehensive dataset documentation, as this evaluation tool section does not directly influence bias creation but significantly aids in its identification.

Drawing from and building on these existing frameworks, the BEAMRAD tool consists of 11 sections (categories) that together list 45 key items (Appendix I). Most of these categories and items are included in the BEAMRAD as they allow us to pay specific attention to parts of dataset documentation that are considered particularly important in the detection of potential sources of bias in a dataset. In what follows we describe the different sections and motivations for including the respective key items. Figure 1 provides an overview of individual sections we included in the dataset evaluation tool and the types of bias that risk emerging if the requested information in that respective category is insufficiently documented.

- **General Description.** This section focuses on questions related to fundamental attributes of datasets such as the description of the primary motivation for creating the dataset, details about its creators and versioning information. Contextual information is important for meaningful interpretation of data^29^, making it vital to report for (potential) data users. A well-crafted description, along with the inclusion of relevant keywords and meaningful titles, significantly enhances the discoverability of datasets for public use. In addition, dataset version control is significant for deep-learning developers as it allows them to track changes in a dataset such as file additions, removals, and modifications of both the data as well as the data annotations. Missing documentation about dataset modifications risks leading to data leakage between training and test sets^30^, or it may lead to the inclusion of data files with varying annotations and preprocessing methods. This can, subsequently, lead to the introduction of various biases including sampling and selection bias, or biases that may emerge from data annotations as the updates on these processes in the additional versions of the dataset are underreported^31–33^.
- **Data usage.** Information on the accessibility and usage of the data is included in the data usage section. Moreover, it contains information abou**t** data licensing and data usage agreements which contain the information if and under what conditions the data can be used or redistributed^34^.
- **Data sources.** This section provides questions regarding the origin of data—where the data was acquired and for what specific research purpose (e.g., as part of a clinical routine or a screening study)—, as well as the acquisition period and the inclusion criteria. Such details are important to include in the documentation of a dataset as they provide insights into the dataset population and deficiencies in this documentation may be the source of various biases. For example, sampling bias may occur when the training data does not represent the target population^31^. Additionally, selection bias and the presence of unknown confounders are considered common sources of dataset bias^11^. Like sampling bias, they risk emerging through the development of imbalanced datasets, and insufficient description of the geographic location of the data acquisition. Missing information on the inclusion criteria for a respective dataset may make it more challenging for data users to detect and possibly mitigate such biases^30,32^.
- **Metadata.** Metadata usually provides information about data subjects, carrying features that reflect particular social characteristics, such as ethnicity, sex, or medical history. The (uneven) distribution of such characteristics is considered a key source of bias in datasets^35^. However, such sources of representation bias are considered difficult to detect and quantify during the construction of a dataset, unless this information has been explicitly collected and included as metadata and documented as such^36^. In addition, metadata on participant demographics is considered valuable as it informs researchers about potential sources of bias, and it also provides them with the relevant information to conduct sub-analyses per demographic group, which can identify biases^35^.

Metadata also involves information on the medical equipment and technical details used for data acquisition. The reporting of information about the devices used in this process is important as bias may emerge because data appearance may vary due to differences in data acquisition protocols^13^. For medical image analysis, for example, image appearance may vary across scanners and image types that rely on image reconstruction choices as well as on protocols that determine the image resolution, the scanning method or contrast enhancement. For ECG data, these details may encompass factors such as sampling frequency and used leads.

- **Sample Size.** This section includes questions on the number of participants included in the dataset, the number of data samples and the number of inclusions of each individual participant. This information is important to include in the documentation of a dataset as insufficient sample sizes make it difficult to train a model on this data and increase the risk of overfitting to unbalanced data samples^10,32,33^. According to Gianfrancesco et al., this may lead to “underestimation” meaning that a model trained on insufficient data is less likely to provide insight into interesting or important cases^32^. Sufficient reporting on sample sizes thus allows data users to detect and potentially mitigate such issues, as well as the representation biases that can emerge from them.
- **Missing Values.** This section covers a question on the documentation of missing values in a dataset. As Rouzrokh et al. explain, missing values may not be uniformly distributed which leads to unintended representation bias in the data. For example, prior research has shown that underrepresented groups may be less likely to provide sensitive information or attend follow-up visits, resulting in a lack of data that models may inadvertently ignore or misinterpret, leading to bias and potential disparities in model outcomes^30^. This, in turn, has major implications for the fairness of models trained on such datasets. As such, it is of critical importance to document missing values in the data to improve the possibilities to detect this type of representation bias and develop strategies to mitigate it.
- **Training & test set distribution.** This section provides questions regarding the partitioning of the data into training and test sets. This information is important to provide in dataset documentation as potential forms of bias may emerge by splitting the data into training and test sets^30^. First, Rouzrokh et al. explain that because medical data such as MRs, CFPs or ECGs are commonly clustered at different levels, data may leak between training and test sets even if developers seek to make sure that data are not repeated in either one of them^30^. Second, the authors observe that imbalanced data distributions may be further amplified through training and test splits. This may lead to the underrepresentation of specific participant groups (e.g., patients with complications) in the test data in comparison to the real-world population. Consequently, it becomes more difficult for researchers to rely on the reported model performance applied to their test set, as the results are less likely to generalize to real-world data^30^.
- **Data annotation.** This section is dedicated to a series of items that focus on the data annotation process. Bias through dataset annotation may stem from several sources, and it is therefore of critical importance to document this process carefully. Kohli et al. describe two principal annotation challenges for biomedical imaging: (1) the question of interoperability and (2) the construction of ground truth^37^. The authors first highlight the absence of universally accepted methodologies for annotating the ground truth of medical images, making it difficult to share and reuse the data for training models across different contexts. Second, the study emphasizes that medicine is a highly ambiguous research field and manual annotations by experts (e.g. radiologists or cardiologists) may not unequivocally represent a ‘ground truth’^37–39^.

In addition, some questions included in the annotation section focus on the disciplinary background of data annotators as well as their years of expertise. As Denton et al.^19^ state, a description of the annotator pool is of critical importance as annotation tasks are subjective and the annotators’ background may contribute to the emergence of bias through labeling ambiguities or the variability between annotators.

Lastly, the section covers a question on the data annotation protocol. According to Rädsch et al. bias in medical image analysis is likely to occur through the data annotation process as “data is typically sparse, inter-rater variability is naturally high, labeling ambiguities occur and medical experts have their individual style of annotations”^20^. Therefore, the provision and documentation of labeling instruction protocols is important to ensure that annotation quality is maintained^20,40,41^. To substantiate their statement, Rädsch et al. explain that this issue has also been addressed by MICCAI in their comprehensive BIAS reporting guidelines^18^ as it “comprises an entire paragraph on reporting the annotation process, including the labeling instructions. Before conducting a competition in the scope of a MICCAI conference, researchers must put the report for their competition online to foster transparency and reproducibility, and to prevent cheating.”^20^. However, their inquiry into the labeling instructions for the datasets underlying all MICCAI competitions officially registered between 2021 and 2023 shows that even though the BIAS guidelines explicitly require a link to the labeling instructions, 76% of the recent MICCAI competitions did not provide sufficient reporting on the data labeling protocol^20^. Following these insights, we included a key item that allows us to evaluate the documentation of labeling instruction protocols for publicly available datasets.

- **Preprocessing.** The documentation of data preprocessing methods and a justification of those methods (e.g., cropping, contrast enhancement) are considered a standard requirement in the BIAS guidelines^18^. For this reason, this category is included in our dataset documentation evaluation tool.
- **Sources of error.** This section focuses on evaluating the reporting of the potential sources of error in a dataset. We include this category in our tool because Maier-Hein et al. state that the comprehensive description of potential sources of error in the data are particularly important to include in the documentation of a dataset as they may emerge through every stage of the dataset construction process^18^. In addition, the authors stress that dataset creators should include reporting on the “magnitude of different error sources” and explain the concepts (e.g., intra-annotator and inter-annotator variability) and methods that have been used to quantify them.

Taken together, the complete version of our dataset documentation evaluation tool (Appendix I) was edited and finalized in close collaboration with a consortium of experts, including medical specialists, AI researchers working in medical domain, expert researchers in Critical Data Studies and AI ethics as well as Health Law.

## Dataset eligibility and selection

To identify a comprehensive set of publicly available datasets and their accompanying documentation to be included in this study, we drafted a series of selection criteria:

1. Through this case-study investigation, we aim to thoroughly assess the present state of dataset documentation and reporting for datasets containing three data types: MRI, CFP, and ECG. These modalities have been selected as examples to qualitatively apply our BEAMRAD evaluation tool. As a first example, we focus on MRI as it is one of the key medical image modalities for which deep-learning models are being extensively developed^41,42^. Beyond radiology, we selected CFP data as example as fundus photography is one of the most widely adopted (non-radiology) imaging modalities, which has also played a significant role in the application of deep-learning systems in medicine because CFP data was used in the first ever FDA-approved AI solution for health decision-making^43^. More specifically, CFP datasets are usually large and challenge data diversity and quality as the data has been collected through screening studies that acquire data at multiple locations (e.g., hospitals, public/private eye clinics or opticians) using a variety of different scanners^21–23^. As deep-learning challenges provide publicly available datasets for research MRI and CFP datasets included in this study were selected from the Grand Challenge platform^44^. Lastly, we focus on ECG data as the number of publicly available ECG datasets is increasingly expanding^25^. ECG is a major diagnostic tool in cardiology. PhysioNet repository, which hosts datasets for common signal modalities such as ECG, electroencephalogram (EEG), and electromyography (EMG), runs an annual event dedicated to only ECG data and challenges in computational cardiology - the George B. Moody PhysioNet Challenges^45^.
2. As this study’s scope aims to examine and evaluate recent medical dataset documentation methods and strategies in MRI CFP and ECG, we only included datasets and accompanying documentations published between January 2019 – June 2023. This date was set as the Biomedical Image Analysis ChallengeS (BIAS) initiative introduced their comprehensive BIAS reporting guidelines^18^ for biomedical image analysis competitions in 2018. These guidelines are expected to have set new standards for reporting on dataset construction for publicly available medical imaging datasets.
3. We only considered datasets that contain novel data. This means that we did not select datasets that have been constructed by combining multiple datasets that have previously been published, also known as remix datasets^11^.

**Fig. 2:**
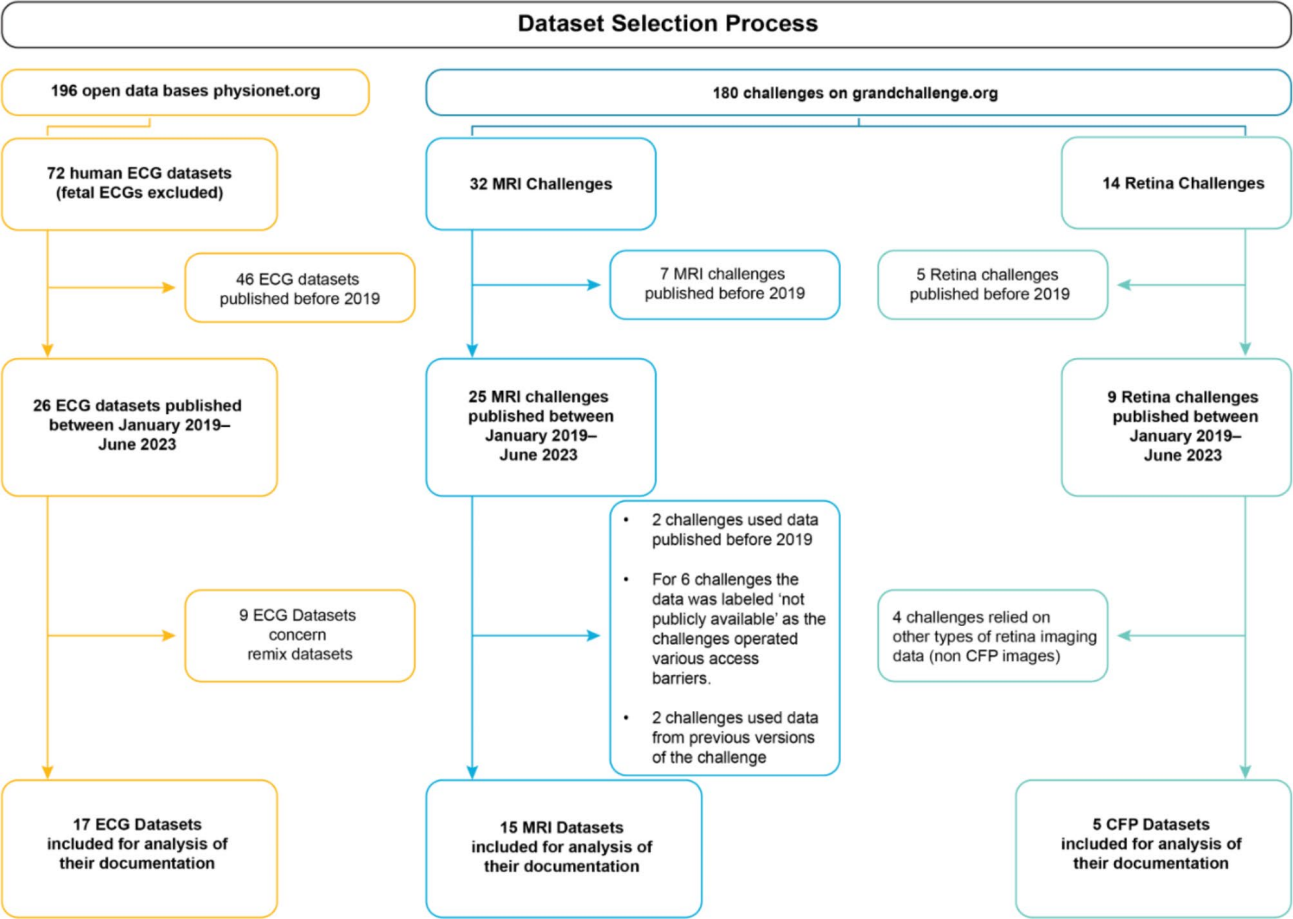
The overview of the dataset selection process.

Following these selection criteria, we finally included a set of 37 medical datasets, comprising 15 MRI datasets, 5 CFP datasets and 17 ECG datasets (Appendix II). This process is visualized in Fig. 2.

## Assessing Dataset Documentation for Medical Imaging and Signal Data

As the aim is to effectively adapt published guidelines that suit various medical data beyond challenge datasets, the questions allow us to systematically evaluate the dataset documentation on multiple aspects of the dataset construction process such as the selection of data sources, metadata, or data annotation. Specifically, we investigate the extent to which researchers follow and interpret dataset documentation guidelines for the construction of datasets in medical imaging (MRI and CFP) and evaluate the current ways in which publicly available ECG datasets are documented. Our evaluations were conducted through a structured content analysis of the selected dataset documentation materials. Following the questions on our pre-developed evaluation tool, our analysis focused on evaluating the aspects dataset authors document about their dataset construction processes and assessing how that information was communicated through their reporting. To do so, we structured the relevant statements per data type and qualitatively coded them according to the list of questions posed in our tool. This allowed for a qualitative assessment and comparison of dataset documentation practices for each data type. Finally, we build on this qualitative content analysis, investigating how the data documentation may contribute to the mitigation of biases in machine-learning models. By employing this research methodology, we comprehensively assess the state-of-the-art medical dataset documentation and aim to better understand how documentation practices can impact the use and re-use of machine-learning solutions.

We present our findings following the overarching themes derived from the BEAMRAD tool we developed for this study. As listed above, these themes include the assessment of the general dataset descriptions as provided in the documentation of the datasets; information provided on data usage; data sources; metadata; data sample sizes and missing data. Furthermore, the results are structured according to the information provided about the data annotation process; training and test distributions of respective medical datasets; as well as the documented information on the relevant sources of relevant sources of error.

**Table 1:**
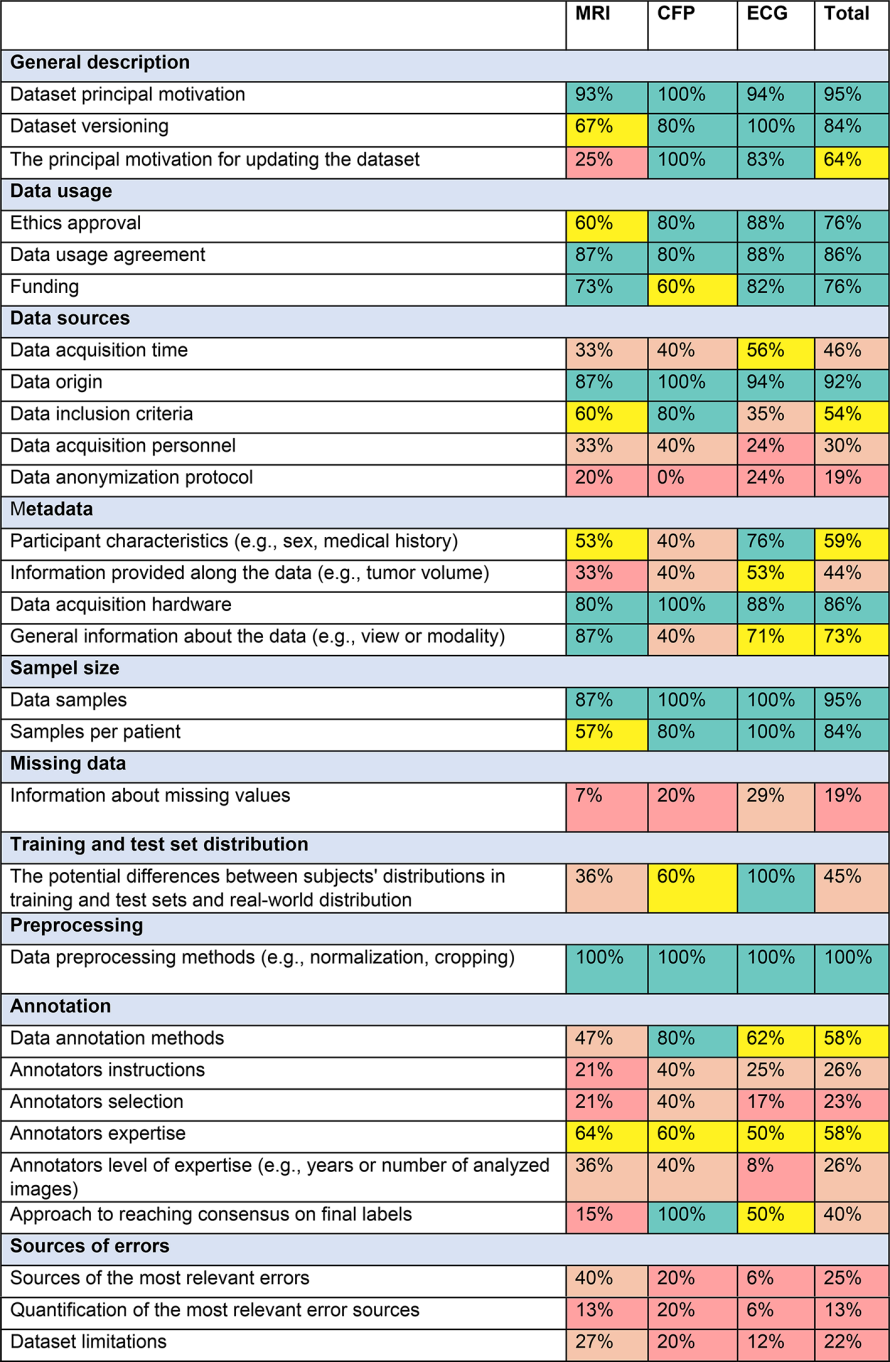
A summary of dataset evaluation outcomes acquired with the BEAMRAD tool, structured for each data type (MRI; CFP; ECG).

The percentages were determined by comparing the number of datasets that provide information on specific dataset documentation points and the total number of datasets for which such information is pertinent. We adhere to the following formula.

$$\:P\:=\:\frac{Numbers\:of\:datasets\:providing\:\text{inf}ormation}{Total\:number\:of\:datasets\:for\:which\:\text{inf}ormation\:is\:relevant}x\:100\%$$

where P represents the percentage as delineated in the table. The denominator of this formula will exclusively include datasets for which pertinent information could be provided. For instance, datasets lacking annotations were excluded from inquiries related to annotations.

## Results

**Fig. 3:**
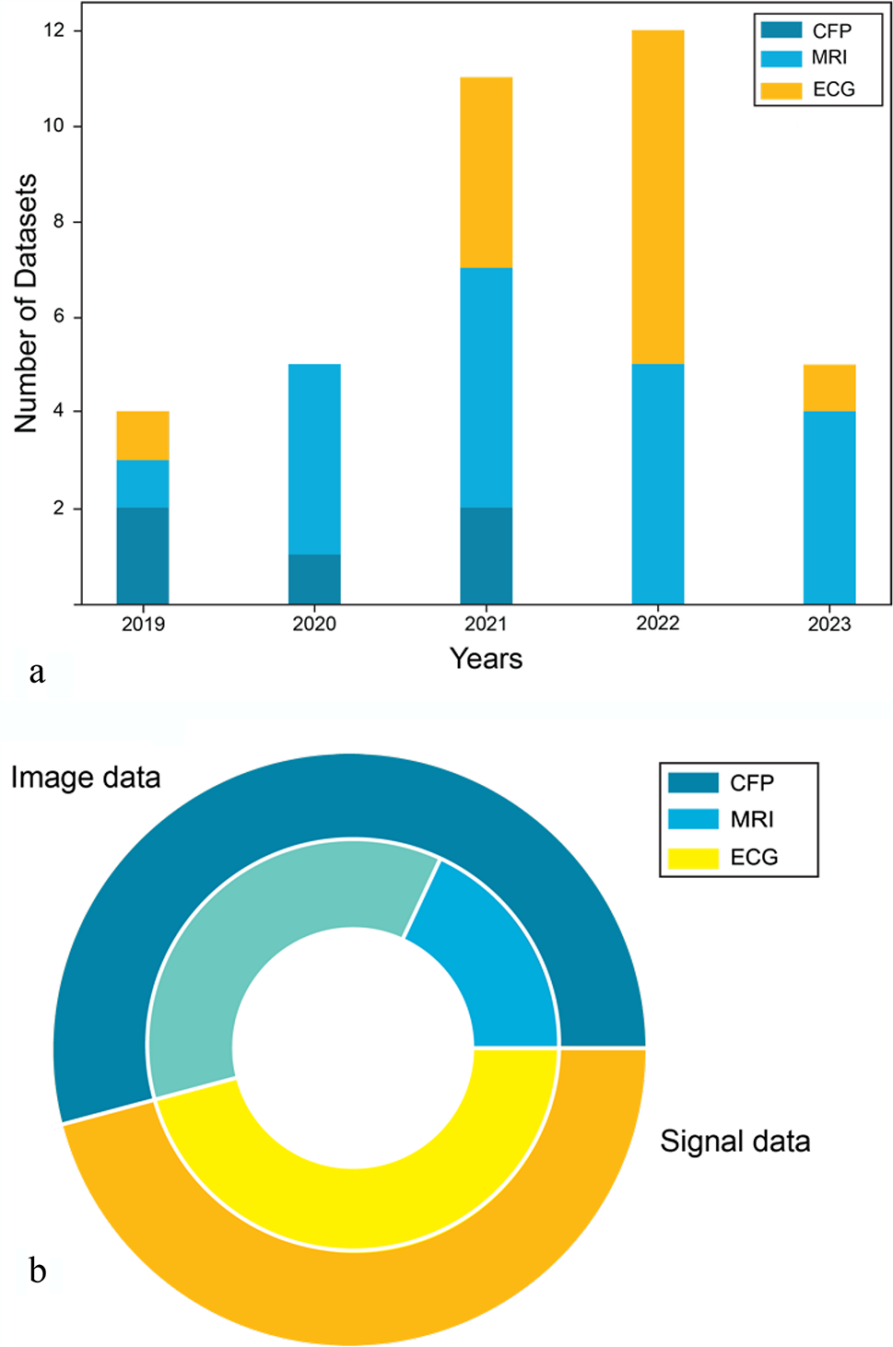
Overview of analyzed datasets, grouped per modality: (**a**) Number of datasets analyzed peryear, (**b**) Proportions of the analyzed dataset number for each modality.

Since 2019, the number of publicly available MRI, CFP and ECG datasets has increased consistently across all datatypes. Figure 3 illustrates the distribution of the 37 analyzed datasets categorized by data type (15 MRI, 5 CFP, 17 ECG) in relation to their respective publication year (a). Additionally, Fig. 3 shows the proportional representation of each data type relative to the total number of included datasets (b). The documentation we encountered ranged from brief notes on websites to solely scientific articles related to a dataset, or comprehensive biomedical imaging challenge documentation. Table 1 outlines the summary of our results, showcasing the percentage of datasets that include relevant information in their documentation to address the questions posed in our evaluation tool. As indicated in the figures (%) presented in the final column (Table 1, ‘Total’), we find a notable proficiency in the provision of information on general dataset description; data usage; details about data origin; acquisition hardware; sample size; and preprocessing techniques (e.g., cropping) across all data types. In contrast, a substantial portion of the documentation we reviewed provides insufficient information concerning missing values, anonymization processes, some aspects of data annotation, and potential sources of error.

Overview of analyzed datasets, grouped per modality: (a) Number of datasets analyzed per year, (b) Proportions of the analyzed dataset number for each modality.

## General description

Out of all the datasets we included in our analysis, 95% provide the motivation and objectives behind the dataset creation. For the datasets that featured multiple versions, 64% articulate the primary rationale for updating the dataset. Notably, CFP and ECG datasets provide comprehensive documentation that details versioning information and the primary motivations behind dataset updates. ECG datasets are available on the Physionet platform^45^, which features a dedicated section for release notes. The Grand Challenge platform^43^ recommends that *“the public training data should be uploaded to a public data host and the secret test data should be uploaded to a private archive on grand-challenge.org”*. Grand Challenge suggests zenodo.org as its primary platform for data hosting, which also provides a section for versioning information. Four out of the five CFP datasets included versioning information. Among these datasets, three opted for zendodo.org as their hosting and publications platform. Only one CFP dataset presented more than one version, accompanied by explicit reasons for the updates. 14 out of 17 MRI datasets published their dataset on zendo.org and 10 datasets provided information on dataset versioning. Four MRI datasets report that they consist of more than one version, but only one of them is accompanied by specific information that provides explicit reasons for the versioning updates.

### Data usage

Table 1 illustrates a consistent trend across data types, with no significant variation in reporting on elements regarding data usage. On average, 76% of analyzed datasets provide information on ethics approval, 86% on data usage agreements, and 76% include details regarding funding and sponsorships.

## Data sources

Our analysis indicates that 34 out of 37 datasets provide information on the respective geographical locations of data collection. However, many dataset descriptions or reporting documents lack specific details about data acquisition dates; only 17 out of 37 datasets include this important information. Notably, among the CFP datasets under study, only two^23,46^ specify the years from which the data originates. However, all fundus imaging datasets provide specific information on data origin. Information about data inclusion criteria was provided by 26 out of 37 dataset documentations. Among the CFP datasets 80% furnished this information. Those datasets originate from screening examinations, adhering to the screening inclusion protocol^23^, or involve individuals who consulted a doctor due to concerns about their symptoms (e.g.,^22,46^) . Due to high costs and specific examination requirements, MRI datasets primarily focus on patients with preexisting medical conditions, such as those with prostate cancer^47^ or ischemic stroke (e.g.,^48^) , and 60% of them provide the specific inclusion criteria. Populations included in ECG studies encompass individuals with specific health conditions (e.g.,^49^) as well as healthy populations (e.g.,^50^) .  However, we find that ECG datasets are less likely to detail participant inclusion criteria as only 30% of the ECG datasets we analyzed report this information through their documentation. This becomes particularly visible for ECG datasets that report including healthy participants.

30% of all datasets explicitly provide information about the individuals responsible for the data acquisition. For 4 ECG datasets, the acquisition personnel comprised a diverse group, including nurses, psychology students, and specialized clinical staff. For 2 CFP datasets that included this information, data collection was performed by primary clinical personnel and ophthalmologists.

Out of 37 examined documentations 7 provide insights into the data anonymization process. Remarkably, none of the CFP datasets includes this information. For all ECG datasets, 4 included information on the anonymization process but this documentation varied from brief mentions that data went through an anonymization process^51,52^, to an explicit indication of the use of a free open-source data anonymization tool, and the description of additional steps to further anonymize any demographic information^53^. Similarly, among the MRI datasets, anonymization information varied from a brief note indicating the anonymization process^54^ to comprehensive descriptions outlining specific steps for anonymization and the utilization of existing protocols^48,55^.

## Metadata

Our review shows that details regarding the inclusion of metadata concerning patient attributes were present in 59% of all datasets we analyzed. Most of these datasets provided significant information on participant demographics, with sex, age, and weight being the most prevalent attributes across all data types. Information provided along the data pertains to any details accompanying the data but excludes patient characteristics. This type of information was present in 40% of the datasets analyzed, being most prevalent among MRI datasets. In the context of MRI datasets, these additional details described parameters such as the number of lesions and lesion sizes in scans (e.g.,^56^). Moreover, supplementary derived information that could be beneficial for segmentation algorithms, such as prostate volume, was provided in radiology reports (e.g.,^47,56^). In the case of ECG datasets, additional notes primarily consisted of remarks on noise or data quality (e.g.,^50,57^) . Among fundus datasets e.g.,^22^ has additional information for each sample, specifically concerning an image acquisition process, including the position and orientation of the patient. 86% of datasets provide details about the devices utilized in data acquisition. Certain datasets present general information about the hardware employed (e.g.,^58^) , while others provide this information for each sample, including details about the MRI scanner vendor and model (e.g.,^47^) . Furthermore, analyzing this documentation we find that 73% of the datasets include comprehensive information on acquisition general attributes. Notably, among the 15 MRI datasets, 13 provide detailed insights into hardware parameters, encompassing information on view or sequence. For ECG data, 12 out of 15 datasets provided additional information such as sampling frequency.

### Sample size and missing data

Evaluating the documentation on sample size and missing data, we found that all ECG datasets provided information about both the number of data samples and the number of samples per included participant. This information is less available in the documentation of MRI and CFP datasets. Respectively 87% (MRI) and 90% (CFP) of these documentations provide insight into the number of data points. In addition, 57% of the MRI datasets and 80% of the CFP datasets report on the number of samples per participant. More significantly, we found out that of 37 datasets, only 5 provide sufficient documented information on the presence of missing data within their datasets. In contrast to these general results, some datasets state the absence of missing data (e.g.,^50^) or annotations (e.g.,^59^) , while one provides a graphical illustration that delineates the dataset population, indicating missing values for each category^25^.

### Training and test set distribution

Reviewing the documented information on training and test distributions, we find that 45% of the analyzed datasets describe the characteristics of subject distribution between the proposed training and test split. For instance, in one dataset data was partitioned into training, validation, and test sets with disease stratification to ensure good representation in all three sets (e.g.,^46,48^). In another example, the dataset creators describe that they followed a specific training-test division to achieve a balanced label distribution as well as an equilibrium in age and sex representation^25^. Lastly, we find dataset descriptions that explain how test sets were composed differently from training and validation sets to facilitate further assessment of models’ generalizability. For example, in one case the test set contained samples from unspecified medical centers that were not present in the training data^48^. In another dataset, the training set comprises both referable and non-referable glaucoma cases, while the test set incorporates ungradable images^23^. According to dataset creators, this replicates real-world deployment scenarios and contributes to the construction of more robust models.

### Annotation

The reviews of the documentation on data annotation show that all CFP datasets incorporate reporting on annotations, predominantly comprising of the descriptions of specific disease labels assigned to each data sample. Similarly, all MRI datasets include annotations, with the majority oriented towards segmentation tasks. In contrast, for ECG datasets, annotations exhibited substantial variability, ranging from annotating QRS complexes (e.g.,^60–62^) to measuring cerebral patient outcomes (e.g.,^49^), while five ECG datasets report that they did not include any annotations. We find descriptions of the methods used for annotation in 58% of the dataset documentations, while 26% of the datasets reported specific annotation protocols. One example of a dataset that included such annotator instructions reports a link to a document outlining a detailed technical and anatomical guideline for manual expert annotations^54^.

The criteria for annotator selection are also not uniformly observed, as only 23% of the evaluated documentation included such information. Most notably, in one case the creators describe a two-step selection process, wherein experienced ophthalmologists were first trained as graders and subsequently underwent examinations^23^. In contrast, datasets related to ECG were the least likely to disclose details about the selection of annotators.

In our study, 58% of the evaluated ECG documentation includes information on the annotators’ backgrounds. Yet, even when there is reporting on this issue, the specificity of the information varies significantly, from simply writing “expert” to reporting on the annotators’ backgrounds and level of expertise (i.e. years of experience), indicated in 26% of ECG data documentations.

Annotation merging is essential when annotations are made by multiple annotators, as conflicting labels may arise. While 40% of datasets document the methodology for achieving consensus, all CFP datasets, where annotations were obtained from more than one expert provide this information. The process is mostly performed by majority voting (e.g.,^63^) , through consensus of the annotators (e.g.,^62^) , or by directing samples with conflicts to professionals with higher expertise (e.g.,^23,46^). Other documentation reveals diverse conflict resolution strategies. For example, potential biases from varied segmentation contouring approaches (i.e., radiographic testing technologist vs. radiation oncologists) were addressed by case randomization based on patient age, gender, and annotation type to maintain a balanced distribution among annotator subgroups. In addition, a researcher, skilled in anatomical structure, manually curated the obtained annotations, correcting segmentation masks^64^.

### Preprocessing

Of 37 datasets included in the analysis, 18 underwent data preprocessing. More specifically, we find that all datasets that incorporated preprocessed data provide comprehensive information on the techniques employed, as well as the specific steps taken to preprocess the data. For ECG common preprocessing steps include noise filtering (e.g.,^7,14^) and resampling (e.g.,^15,20^). For other data types, this process includes cropping (e.g.,^42^) , reslicing (e.g.,^32^), and defacing for MRI (as a deanonymization step) (e.g.,^6,8,32^) .

### Sources of error

Our results (Table 1) show that the provision and quantification of sources of errors through data annotations and the description of specific dataset limitations are not part of regular dataset documentation practices. Only 7 out of the 20 image datasets (MRI and CFP) provide information on the potential sources of error through dataset annotations. For the ECG datasets we reviewed, 1 out of 17 datasets reported this information. More generally, 4 out of all datasets reported on the quantification of the most relevant sources of error, and 7 datasets were accompanied by information detailing their limitations.

Documentation from MRI studies, we find, proved to be the most informative concerning the most significant error sources in annotations. For example, some datasets highlight potential errors arising from poor image quality and potential artifacts, influencing the confident identification of clear boundaries^65,66^.

Reporting on the quantification of relevant error sources is not a widespread practice in the assessed documentation. Nonetheless, we find a few examples of good practices, including the description of the use of the Dice coefficient as a metric to assess delineation overlaps among raters^48^ or a median of an absolute error between experts^60,61^. Another dataset provides a detailed description of the results of annotating nine samples by three annotators^54^. The organizers of one challenge report that, due to the time-intensive labeling process, the dataset creators selected only two cases to evaluate the intra-/inter-reproducibility of manual annotations conducted by a highly experienced reviewer^65^. Lastly, another example dataset reports annotators were not only assessed at the start but were also periodically monitored throughout the data grading process. If their performance dropped below a certain threshold, they were excluded from the study. Subsequently, all images they labeled underwent re-grading by any of the remaining annotators^23^.

We infrequently observe disclosures of dataset limitations in dataset documentation practices. The following shortcomings were given: (1) potential constraints associated with data acquisition and image registration^49,66^; (2) limitations arising from diverse modalities and scanner settings^55^; (3) The complex nature of medical issues, and the absence of relevant clinical context in the respective datasets^49^; (4) The lack of diversity in a dataset, and the importance to further improve it^23^.

### Discussion and Conclusion

Over the last years, a growing number of scholars have highlighted the necessity for comprehensive documentation of both datasets and models to identify and address potential biases^13–15^. This self-evaluation of datasets provides directions and illustrates best practices aimed at bias reduction and consideration of model limitations, including the scope of its applicability^11^.

This study focuses on mitigating of bias resulting from dataset development practices by introducing the BEAMRAD tool, which supports the completeness of medical dataset documentation to facilitate the prevention, identification, and mitigation of potential biases arising from dataset construction. We examined the existing documentation standards for medical datasets. Our analysis specifically targeted MRI and CFP as representative of image modalities and ECG as an example of signal data. During our study, we reviewed the documentation quality for these datasets. Analyzed image datasets originate from the Grand Challenge platform, all released post-2018, after the publication of BIAS guidelines^18^. The signal datasets are sourced from the PhysioNet platform^45^, where we encountered a lack of specific guidelines for the descriptions of the datasets published on this platform. Even though the existing reporting guidelines have only been developed for datasets used for medical image analysis^10,18^, we do not find a significant difference in the amount of documented information for publicly available image datasets (MRI and CFP) or signal datasets (ECG). When examining the results related to data annotation for CFP and MRI datasets from the same platform, however, it becomes evident that documentation is more comprehensive for CFP datasets. This might suggest that, despite the existence of reporting guidelines, the adherence to and interpretation of these guidelines by individual dataset creators is perhaps even more important. To advance current practices, we argue for dataset documentation to become an iterative process, conducted in parallel to the creation of the datasets themselves.

The BEAMRAD tool presented in this paper provides a guide to secure such comprehensive reporting. Looking forward, we note two other incentives or strategies to further improve the community’s adherence to existing guidelines for dataset documentation such as BIAS. First, as Rädsch et al. explain, the MICCAI special interest group for challenges in biomedical imaging is already considering stricter rules for reporting to ensure and improve data quality for challenges^20^. Second, we argue that there is a critical role for community-based platforms or repositories like Grand Challenge and PhysioNet to consider stricter rules for dataset creators to follow guidelines in order to publish a dataset through their outlets.

Furthermore, our findings highlight a series of gaps in dataset documentation as they indicate that detailing and quantifying sources of annotation errors and describing the limitations of datasets remains a minority in medical dataset documentation. It is paramount to emphasize that incomplete or absent documentation, particularly concerning aspects such as data annotation and participant inclusion criteria, can result in the training of highly biased models using such datasets (e.g.,^30–32^). This can eventually even affect a model’s robustness, in terms of how well it can maintain its performance in various contexts. Consequently, these models risk becoming less suitable for further clinical application and complicate the possibility of adapting them into clinical practice^11^. In our study, we explicitly outlined the types of biases that may arise from incomplete and insufficiently detailed information within various aspects of dataset documentation. The direct enumeration of potential biases underscores the risks associated with specific information absence, providing crucial insights for data users.

Dataset creators play a pivotal role in ensuring data accuracy, completeness, and relevance. Their expertise helps to capture nuanced dataset information, address potential biases, and document crucial details. Reliable and well-curated medical datasets are essential for developing accurate and effective machine-learning models, diagnostic tools, and healthcare applications. While data quality is indisputably a paramount requirement for constructing a medical diagnostic system, other facets of the model-building process are also susceptible to biases^11^. As emphasized in datasheets for datasets, guidelines, and checklists serve as valuable tools that facilitate reflection on the dataset creation process. This reflective practice promotes transparency, and thoroughness, and is deemed instrumental in producing more replicable datasets. Even though authors of datasets may employ diverse guidelines or templates, the primary objective is to critically engage with and reflect on dataset-creation practices by ensuring all the relevant information we listed in our tool is included in the main documentation of a dataset. Such documented reflections are important as data scientists’ discretionary decisions throughout these processes can influence model bias and transparency^67^. Reflection thus involves providing information related to the general description of the data; data usage; data sources; metadata; sample sizes; data distribution; missing data; annotation characteristics; data preprocessing; sources of errors; and data limitations. Metadata documentation on participant demographics, for example, informs researchers about potential sources of bias, but also allows for performance sub-analyses by demographic group, which can further identify biases^35^. However, the sharing of metadata must strictly comply with GDPR^68^ and other applicable privacy regulations to ensure the protection of participant information. Our evaluation tool provides a way to actively engage with and assess the documentation of a dataset concerning the specific sections listed above. To meaningfully account for potential sources of bias in the data, it is crucial to have comprehensive information and justification documented accompanying a public dataset. The evaluation tool can guide dataset creators in documenting the dataset by answering its consecutive questions or it can function as a checklist to ensure all the vital information is included in the previously created documentation of a dataset. In the section ‘sources of error,’ for example, creators are directly prompted to describe and quantify potential sources of errors and limitations of their respective datasets.

Model developers are responsible for creating the algorithms powering healthcare applications. Their expertise is fundamental in creating models that can analyze medical data, make predictions, assist in diagnostics, and contribute to personalized treatment plans. Dataset curations play a pivotal role in model development, impacting possible biases and solution quality. This stage is crucial for guaranteeing the accuracy, robustness, and generalizability of models, thereby enhancing their potential for subsequent clinical application. Models possessing these attributes are more likely to be effective in clinical practice. Comprehensive dataset documentation aids data users in choosing the most fitting datasets for their project goals efficiently. Furthermore, validating created solutions on externally curated datasets that closely represent clinical use cases is important. This approach opens opportunities for a smoother translation from research to clinical application^11^. The BEAMRAD tool offers key insights throughout model development, helping to create better models. It allows to assess dataset representativeness during the design phase, ensuring models are built with data that reflect the target population. Information regarding dataset sourced from BEAMRAD tool can moreover help to navigate verification if model is working well during the development and implementation phase by supporting developers in identifying errors stemming from datasets. A notable example of lacking data transparency occurred during the COVID-19 pandemic, when numerous biases were exposed in machine learning models, particularly due to the use of “Frankenstein” datasets—datasets assembled from overlapping sources, leading to inflated performance metrics^16^.

### Limitations

This paper focused on evaluating the documentation of datasets containing three data types (MRI, CFP, and ECG), accessible on two platforms: Grand Challenge and PhysioNet. In this study we selected a number of datasets to demonstrate the BEAMRAD tool. While we present evaluation results by data type (Table 1), the inclusion and comparison of imaging data from challenges (MRI and CFP) and open datasets (ECG) is limited. Further research should investigate whether the findings generalize to other data types and data collected outside of challenges. Furthermore, this research does not detail the documentation quality for datasets containing other data types such as X-ray, Computed Tomography (CT) or Electroencephalogram (EEG). To further expand and build upon this work, we outline suggestions for further research in the next section.

### Further research

Future investigations could consider treating documentation guidelines as dynamic, adaptable resources that can evolve with emerging data types. Engaging expert focus groups offer the potential to fine-tune and enhance dataset documentation evaluation tools, tailoring them to specific medical domains or contexts. Additionally, we suggest gathering user feedback through workshops and conferences for iterative improvements to ensure the continued effectiveness of these tools in addressing real-world challenges. This ongoing research aims to refine dataset documentation practices for diverse applications. Furthermore, our results highlight the relative scarcity of reporting on data limitations, annotation errors and their quantification, even though dataset documentation guidelines^10,15,34^ have explicitly called for this information to be reported. Our observations thus underscore the necessity for further exploration in this direction, emphasizing the development of detailed standardized rules and recommendations for researchers to document their datasets. In parallel, the approach towards bias mitigation we present in this paper can further be substantiated by other frameworks such as causal reasoning, which have proven to improve transparency on decisions about dataset construction and processing while also providing detailed categorizations of potential biases and mitigation techniques^69^. Future research could investigate the intersection of biases from data construction and documentation with existing detection methodologies, while also reviewing bias mitigation tools that address biases linked to dataset development practices to provide systematic solutions. An especially interesting direction would be to explore quantitative components that identify bias and measure the impact of improved documentation on model performance and its effects for clinical decision-making. Summarizing documentation practices for other dataset types, such as electronic health records—which often have restricted access—would also be highly beneficial. Additionally, a longer-term goal should involve evaluating the tool’s effectiveness on model development, allowing for an assessment of BEAMRAD’s impact over time as more datasets adopt its documentation standards.

With this paper, we have contributed a comprehensive framework for evaluating the quality of dataset documentation, a framework that extends beyond image or challenge data domains. Scientific resources such as public medical datasets are becoming increasingly available, as open-science principles emphasize the need for data sharing and improving the accessibility of models. Therefore, it is important to evaluate the quality of these datasets, encompassing not only the inherent data quality but also the quality of documentation associated with the dataset. The main goal of establishing a comprehensive dataset documentation is to develop a valuable resource that helps creators and users of datasets identify potential bias sources. The BEAMRAD tool we developed and applied in this research acts as a discerning lens and provides a focused perspective on bias origins that have a significant impact on a model’s abilities to make suitable classifications and predictions and mitigate the risks to amplify existing social inequalities and discrimination, which influence the generalizability and applicability of models for further clinical applications. As such, this approach holds unique value, rooted in an interdisciplinary team of experts in medical AI, Critical Data Studies and AI ethics, medical specialists, as well as Health Law.

## Electronic Supplementary Material

Below is the link to the electronic supplementary material.

Supplementary Material 1

## Data Availability

The datasets used and/or analysed during the current study available from the corresponding author on reasonable request.
