# Supplementary material for: Assessing the documentation of publicly available medical image and signal datasets and their impact on bias using the BEAMRAD tool
Source: Sci Rep. 2024 Dec 30;14:31846. doi: 10.1038/s41598-024-83218-5 (PMC11686007; doi:10.1038/s41598-024-83218-5)
Supplement: Supplementary file 1 — Supplementary Material 1 [file 41598_2024_83218_MOESM1_ESM.docx]

**Appendix I — BEAMRAD Tool: Bias Evaluation And Monitoring for Transparent And Reliable Medical Datasets**

Overview of the dataset documentation evaluation tool.

| **Category** | **Key Items** |  |
| --- | --- | --- |
| **Title** | 1. **Is there a title/acronym available for the dataset?**  If yes, please provide the title of the dataset.      1. **Is there information on how to cite the resource?**  If yes, provide the citation. |  |
| **General description** | 1. **Please describe the target entities,** i.e., the anatomical region of subjects from whom the data was acquired (e.g., brain shown in MRI). 2. **What data type is included in this dataset?**  Image data such as MRI or Color Fundus Photography (CFP), or signal data (ECGs)? 3. **Is the principal motivation and objective for the dataset described?**  If so, please provide a summary of the principal motivations behind the development of the dataset. 4. **Are there different versions of this dataset?**  If so, is the principal motivation and objective for updating this dataset described? Please, provide the current version and a description of the principal motivations. 5. **Provide keywords for the dataset** (e.g., datatype, modality, body part, relevant medical terms). 6. **Is there information about coordinators or organizers involved in the dataset creation? Is there a list of contributors available?**  If so, please provide the list of contributors, including their affiliations. 7. **When was the dataset published (first version)?** Please provide the date of publication (year, month). 8. **Is the dataset associated with a challenge?** If so, please provide the challenge name. |  |
| **Dataset usage** | 1. **Is there an official website or other place where the dataset is hosted?** If so, please provide the location (e.g., a URL). 2. **What is the accessibility of the dataset** (choose from provided  categories) [5]? Are there any specific barriers or formal agreements?  If yes, provide details on them.      1. Open access – no requirements for access or only sign-in    2. Open access with barriers – datasets fulfilling the theoretical criteria for open access, but are inaccessible because of unpredictable reasons (e.g., no response to requests or broken hyperlinks)    3. Regulated access – required the fulfilment of formal agreements or approvals. 3. **Is there information about the ethics approval?**  If so, please provide the reference to the ethics approval or waiver. 4. **Is there a data usage agreement? Is there an explicit listing of the license applied?** If so, please describe the license. 5. **Is there information regarding sponsoring/funding provided?**  If so, please provide the sponsorship information. 6. **Are there any publication restrictions?**  If so, please provide the restriction information |  |
|  |  |  |
| **Data sources** | 1. **Is there a description of when the data included in the dataset was acquired (e.g., between 2005-2020)?** If so, please provide the information. 2. **Is there information about the origin of data** **(e.g., country and hospital)?** If so, please provide this information. 3. **Is it a single-center or multi-center study?** 4. **Are the criteria for data inclusion described? (e.g., consecutive participants, inclusion and exclusion criteria)**. If yes, please provide the description. 5. **Are there relevant characteristics (e.g., level of expertise) of the subjects (e.g., surgeon)/objects (e.g., robot) involved in the data acquisition process?** If so, please provide this information. 6. I**s there any information on the data anonymization protocol?**  If so, please describe the anonymization protocol. |  |
| **Metadata** | 1. **Is there information about the participant (e.g., age, medical history)?**  **If relevant, what are the features, variable types, and the number of categories provided for the variable?** Please provide a description. 2. **Is any information provided along with the data?** If yes, what kind of information? 3. **Is there information about the hardware used for the data acquisition?**  If so, provide the information about the hardware—i.e. the vendor and the specific model. 4. **Is there general information about the data acquisition protocol, such as view or modality?** If so, what kind of information is provided? |  |
| **Sample size** | 1. **What is the number of participants and the number of data samples?**  Please, provide the number of samples. 2. **If there is more than one inclusion for a participant, what is the number of data samples per participant?** Please, provide the number of samples per participant |  |
| **Missing data** | 1. **Is the information about missing values provided?**  If so, please describe the details. |  |
| **Training and test set distribution** | 1. **Is data divided into training/test sets?**  If the dataset consists of training/test sets, how is the data distributed? Please provide a description. 2. **Are both the training and test datasets available?** 3. **Is there an explanation for the potential differences between distributions of the data subjects in in training and test sets and the real-world distribution?** |  |
| **Data Annotation** | 1. **What types of annotations are included in this dataset?** If so, please describe the different types of annotations. 2. **Are the data annotation methods explained?** If so, please describe the methods. 3. **Are there human annotators involved?** 4. **If human annotators were involved, is the annotation instruction protocol available?** If so, please describe the protocol. 5. **If human annotators were involved, is the number of annotators indicated? If yes, how many individual annotators are involved in the annotations process?** Please provide this information. 6. **If human annotators were involved, is there information on how they were selected?** If so, please provide this information on the selection process. 7. **If human annotators were involved, is the disciplinary background of the annotators indicated, including their years of expertise?** If so, please provide this information. 8. **If there are multiple annotations, is the approach to reaching a consensus on the final labels described by the dataset’s creators?** If so, provide this information. |  |
| **Data preprocessing** | 1. **Is the data preprocessed? If yes, how was the data preprocessed?** Please describe the methods (e.g., cropping, resampling, and contrast enhancement). |  |
| **Sources of error** | 1. **Are the most relevant possible sources of error (e.g., error through annotation variability) described in the documentation of the dataset?** If so, please provide this information. 2. **Are the most relevant error sources quantified?** (For instance, by giving specific information for training, validation and test cases?). If so, please provide a list of the quatifications. 3. **Is there information regarding the limitations of the dataset?** If so, please provide the information. |  |

**Appendix II – Tables of included datasets [MRI, CFP, ECG]**

| **Magnetic Resonance Imaging** | | | | |
| --- | --- | --- | --- | --- |
| **Title** | **Challenge** | **Dataset** | **Documentation** | **Challenge Status [Open / Closed] + Data Access Date** |
| BONBID-HIE 2023 - BOston Neonatal Brain Injury Dataset for Hypoxic Ischemic Encephalopathy | <https://bonbid-hie2023.grand-challenge.org/> | <https://zenodo.org/record/8104103> | <https://www.biorxiv.org/content/10.1101/2023.06.30.546841v1.full.pdf> | Closed; Data was accessed August 24, 2023 |
| BrainPTM 2021 - Brain Pre-surgical white matter Tractography Mapping Challenge | <https://brainptm-2021.grand-challenge.org/> | <https://zenodo.org/records/6359760> | <https://zenodo.org/records/6359760> | Closed; Data was accessed September 6, 2024 |
| Carotid Artery Vessel Wall Segmentation Challenge 2021 | <https://vessel-wall-segmentation.grand-challenge.org/> | https://vessel-wall-segmentation.grand-challenge.org/ | <https://zenodo.org/record/4575301> | Closed; Data was accessed 14 August 2024 |
| Chaos 2019 - Combined (CT-MR) Healthy Abdominal Organ Segmentation | <https://chaos.grand-challenge.org/> | <https://zenodo.org/record/3431873> | <https://arxiv.org/pdf/2001.06535.pdf> | Closed; Data was accessed August 21, 2023 |
| COSMOS 2022 - CarOtid vessel wall SegMentation and atherosclerOsis diagnosiS challenge | <https://vessel-wall-segmentation-2022.grand-challenge.org/> | <https://zenodo.org/record/6481870> | <https://vessel-wall-segmentation-2022.grand-challenge.org/data/> | Closed; Data was accessed August 23, 2023 |
| CROSSMODA 2022 - Cross-Modality Domain Adaptation for Medical Image Segmentation | <https://crossmoda2022.grand-challenge.org/> | <https://zenodo.org/record/4662239> | <https://arxiv.org/abs/2201.02831> | Closed; Data was accessed August 21, 2023 |
| FETA 2022 - Fetal Tissue Annotation and Segmentation Challenge (FeTA), MICCAI 2022 | <https://feta.grand-challenge.org/> | <https://feta.grand-challenge.org/> | <https://zenodo.org/record/6683366> | Closed; Data was accessed August 14, 2023 |
| HaN-Seg 2022 - The head and Neck organ-at-risk CT & MR segmentation challenge | <https://han-seg2023.grand-challenge.org/> | <https://zenodo.org/record/7442914#.ZBtfBHbMJaQ> | <https://aapm.onlinelibrary.wiley.com/doi/full/10.1002/mp.16197?af=R> | Closed; Data was accessed August 22, 2023 |
| ISLES 2022 - Ischemic Stroke Lesion Segmentation Challenge | <https://isles22.grand-challenge.org/> | <https://zenodo.org/record/7960856#.ZK5or-xBzmE> | <https://www.nature.com/articles/s41597-022-01875-5> | Closed; Data was accessed August 24, 2023 |
| PI-CAI 2022 – Artificial intelligence and radiologist at prostate cancer Detection in MRI | <https://pi-cai.grand-challenge.org/> | <https://zenodo.org/record/6624726> | <https://zenodo.org/record/6522364> | Closed; Data was accessed August 22, 2023 |
| QUBIC 2021 - Quantification of Uncertainties in Biomedical Image Quantification Challenge | <https://qubiq21.grand-challenge.org/> | <https://syncandshare.lrz.de/getlink/fi7wpcsxhK9AjyjBU7vj5y8m/training_data_v3_QC.zip> | <https://syncandshare.lrz.de/getlink/fi7wpcsxhK9AjyjBU7vj5y8m/training_data_v3_QC.zip> | Closed; Data was accessed August 21, 2023 |
| Shifts 2022 - Shifts Multiple Sclerosis Lesion Segmentation Dataset | <https://shifts.grand-challenge.org/> | <https://zenodo.org/record/7051658> & <https://zenodo.org/record/7051692> | <https://arxiv.org/abs/2206.15407> | Closed; Data was accessed August 22, 2023 |
| SynthRAD 2023 - synthetizing computed tomography for radiotherapy | <https://synthrad2023.grand-challenge.org/> | <https://zenodo.org/record/7260705> | <https://doi.org/10.1002/mp.16529> | Open; Data was accessed August 14, 2024 |
| SPIDER 2023 - Spine Segmentation: Discs, Vertebrae and Spinal Canal | <https://spider.grand-challenge.org/> | <https://zenodo.org/record/8009680> | <https://arxiv.org/pdf/2306.12217.pdf> | Open; Data was accessed August 14, 2023 |
| VALDO 2021 - Where is VALDO - Vascular Lesions Detection Challenge | <https://valdo.grand-challenge.org/> | <https://zenodo.org/record/4687995> | <https://zenodo.org/record/4687995> | Open; Data was accessed August 24, 2023 |

| **Color Fundus Photography** | | | | |
| --- | --- | --- | --- | --- |
| **Title** | **Challenge** | **Dataset** | **Documentation** | **Challenge Status [Open / Closed, Date Accessed]** |
| AIROGS 2022 - Artificial Intelligence for RObust Glaucoma Screening Challenge | <https://airogs.grand-challenge.org/> | <https://zenodo.org/record/5793241> | <https://arxiv.org/pdf/2302.01738.pdf> | Closed; Data was accessed September 6, 2023 |
| ODIR 2019 - Peking University International Competition on Ocular Disease Intelligent Recognition | <https://odir2019.grand-challenge.org/> | <https://www.kaggle.com/datasets/andrewmvd/ocular-disease-recognition-odir5k> | <https://www.kaggle.com/datasets/andrewmvd/ocular-disease-recognition-odir5k> | Open; Data was accessed September 11, 2023 |
| PALM 2019 - Pathologic Myopia Challenge | <https://palm.grand-challenge.org/> | <https://palm.grand-challenge.org/> | [https://arxiv.org/pdf/2305.07816.pdf](https://www.google.com/url?q=https://arxiv.org/pdf/2305.07816.pdf&sa=D&source=editors&ust=1726221366564905&usg=AOvVaw31CGdMXazT0xxid0pHPLJr) | Closed; Data was accessed September 14, 2023 |
| REFUGE 2020 - Retinal Fundus Glaucoma Challenge Edition 2 | <https://refuge.grand-challenge.org/> | <https://zenodo.org/record/3714947> | <https://zenodo.org/record/3714947> | Closed; Data was accessed September 12, 2023 |
| RFMiD 2021 - Retinal Image Analysis for multi-Disease Detection Challenge | https://riadd.grand-challenge.org/ | <https://zenodo.org/record/7505822> | <https://www.mdpi.com/2306-5729/6/2/14> | Closed; Data was accessed September 12, 2023 |

| **Electrocardiogram** | | | |
| --- | --- | --- | --- |
| **Title** | **PhysioNet** | **Documentation** | **Data Access Date** |
| A large scale 12-lead electrocardiogram database for arrhythmia study | <https://physionet.org/content/ecg-arrhythmia/1.0.0/> | <https://www.nature.com/articles/s41597-020-0386-x> | August 14, 2023 |
| Autonomic Aging | <https://physionet.org/content/autonomic-aging-cardiovascular/1.0.0/> | <https://www.nature.com/articles/s41597-022-01202-y> | August 14, 2023 |
| Brno University of Technology ECG Quality Database (BUT QDB) | <https://physionet.org/content/butqdb/1.0.0/> | <https://physionet.org/content/butqdb/1.0.0/> | August 18, 2023 |
| Brno University of Technology Smartphone PPG Database (BUT PPG) | <https://physionet.org/content/butppg/1.0.0/> | <https://www.ncbi.nlm.nih.gov/pmc/articles/PMC8440059/> | August 18, 2023 |
| Electrocardiogram, skin conductance and respiration from spider-fearful individuals watching spider video clips | <https://physionet.org/content/ecg-spider-clip/1.0.0/> | <https://physionet.org/content/ecg-spider-clip/1.0.0/> | August 21, 2023 |
| EPHNOGRAM: A Simultaneous Electrocardiogram and Phonocardiogram Database | <https://physionet.org/content/ephnogram/1.0.0/> | <https://www.biorxiv.org/content/10.1101/2021.05.17.444563v2.full.pdf> | August 21, 2023 |
| Haaglanden Medisch Centrum sleep staging database | <https://physionet.org/content/hmc-sleep-staging/1.1/> | <https://physionet.org/content/hmc-sleep-staging/1.1/> | August 21, 2023 |
| I-CARE: International Cardiac Arrest REsearch consortium Database | <https://physionet.org/content/i-care/2.0/> | <https://www.researchgate.net/publication/373478395_The_International_Cardiac_Arrest_Research_I-CARE_Consortium_Electroencephalography_Database> | August 22, 2023 |
| Icentia11k Single Lead Continuous Raw Electrocardiogram Dataset | <https://physionet.org/content/icentia11k-continuous-ecg/1.0/> | <https://www.cinc.org/2021/Program/accepted/229_Preprint.pdf> | August 22, 2023 |
| Influence of the MHD effect on 12-lead and 3-lead ECGs recorded in 1T to 7T MRI scanners | <https://physionet.org/content/mhd-effect-ecg-mri/1.0.0/> | <https://www.cinc.org/archives/2017/pdf/132-090.pdf> | August 22, 2023 |
| Lobachevsky University Electrocardiography Database | <https://physionet.org/content/ludb/1.0.1/> | <https://physionet.org/content/ludb/1.0.1/> | August 22, 2023 |
| Norwegian Endurance Athlete ECG Database | <https://physionet.org/content/norwegian-athlete-ecg/1.0.0/> | <https://www.ncbi.nlm.nih.gov/pmc/articles/PMC9829117/> | August 24, 2023 |
| PTB-XL - a large publicly available electrocardiography dataset | <https://physionet.org/content/ptb-xl/1.0.3/> | <https://www.nature.com/articles/s41597-020-0495-6> | August 24, 2023 |
| Simultaneous physiological measurements with five devices at different cognitive and physical loads | <https://physionet.org/content/simultaneous-measurements/1.0.2/> | <https://journals.plos.org/plosone/article?id=10.1371/journal.pone.0274994> | August 24, 2023 |
| VitalDB - a high-fidelity multi-parameter vital signs database in surgical patients | <https://physionet.org/content/wctecgdb/1.0.1/> | <https://www.mdpi.com/2075-1702/4/4/18> | August 24, 2023 |
| Wilson Central Terminal ECG Database | <https://physionet.org/content/wearable-exercise-frailty/1.0.0/> | <https://ieeexplore.ieee.org/document/9795954> | August 24, 2023 |
